# The Spectroscopic Properties and Microscopic Imaging of Thulium-Doped Upconversion Nanoparticles Excited at Different NIR-II Light

**DOI:** 10.3390/bios11050148

**Published:** 2021-05-10

**Authors:** Tingting Peng, Rui Pu, Baoju Wang, Zhimin Zhu, Kai Liu, Fan Wang, Wei Wei, Haichun Liu, Qiuqiang Zhan

**Affiliations:** 1 Centre for Optical and Electromagnetic Research, Guangdong Provincial Key Laboratory of Optical Information Materials and Technology, South China Academy of Advanced Optoelectronics, South China Normal University, Guangzhou 510006, China; tingting.peng@coer-scnu.org (T.P.); rui.pu@coer-scnu.org (R.P.); baoju.wang@m.scnu.edu.cn (B.W.); zhimin.zhu@coer-scnu.org (Z.Z.); 2 State Key Laboratory of Rare Earth Resource Utilization, Changchun Institute of Applied Chemistry, Chinese Academy of Sciences, Changchun 130022, China; kai.liu@ciac.ac.cn (K.L.); wangfan@ciac.ac.cn (F.W.); 3 MOE & Guangdong Provincial Key Laboratory of Laser Life Science, Guangzhou Key Laboratory of Spectral Analysis and Functional Probes, College of Biophotonics, South China Normal University, Guangzhou 510006, China; weiwei@scnu.edu.cn; 4 Experimental Biomolecular Physics, Department of Applied Physics, Royal Institute of Technology (KTH), SE-106 91 Stockholm, Sweden; haichun@kth.se; 5 National Centre for International Research on Green Optoelectronics, South China Normal University, Guangzhou 510006, China

**Keywords:** upconversion nanoparticles, near-infrared-II, excitation mechanisms, luminescence quenching, microscopic imaging

## Abstract

Lanthanide-doped upconversion nanoparticles (UCNPs) are promising bioimaging nanoprobes due to their excellent photostability. As one of the most commonly used lanthanide activators, Tm^3+^ ions have perfect ladder-type electron configuration and can be directly excited by bio-friendly near-infrared-II (NIR-II) wavelengths. Here, the emission characteristics of Tm^3+^-doped nanoparticles under laser excitations of different near-infrared-II wavelengths were systematically investigated. The 1064 nm, 1150 nm, and 1208 nm lasers are proposed to be three excitation strategies with different response spectra of Tm^3+^ ions. In particular, we found that 1150 nm laser excitation enables intense three-photon 475 nm emission, which is nearly 100 times stronger than that excited by 1064 nm excitation. We further optimized the luminescence brightness after investigating the luminescence quenching mechanism of bare NaYF_4_: Tm (1.75%) core. After growing an inert shell, a ten-fold increase of emission intensity was achieved. Combining the advantages of NIR-II wavelength and the higher-order nonlinear excitation, a promising facile excitation strategy was developed for the application of thulium-doped upconversion nanoparticles in nanoparticles imaging and cancer cell microscopic imaging.

## 1. Introduction

Lanthanide-doped upconversion nanoparticles (UCNPs) are of particular attraction as a kind of luminescent nanomaterials because of their significant advantages over other luminescent probes, such as long lifetime, sharp-band emission, and tunable emissions [1]. Due to their unique photon upconversion ability and ultrahigh photostability, UCNPs attract much attention in the realm of biophotonics and nanophotonic, especially for biological imaging in cells and deep tissues [2,3]. As one of the most commonly used activators in upconversion luminescence systems, Tm^3+^ ion features typical step-by-step upconversion excitation characteristics and rich cross-relaxation (CR) processes, giving birth to multicolor emissions. Therefore, its optical properties can be easily regulated by optical control and chemical synthesis methods, holding great potential in applications such as stimulated emission depletion (STED) super-resolution imaging [4], optogenetics [5,6], and early stage tumor therenostics [7] in nanomedicine [8].

According to recent studies, Tm^3+^ ions can directly absorb photon in near-infrared-II spectral range (1000–1400 nm) without the aid of sensitizers for upconversion luminescence, which is of great significance for application in deep tissue imaging [9,10,11]. Previously, some works have reported upconversion mechanism for Tm^3+^-doped materials under NIR-II wavelengths [12,13], e.g., the energy-looping upconversion nanoparticles under 1064 nm excitation employed for deep tissue imaging [14]. However, these works only concentrated on emissions originating from lower-lying states (e.g., the two-photon 808 nm emission), but ignored the significance of higher-order emissions. On the other hand, for most of commercially available detectors, the sensitivity for 808 nm emission band is not pretty good as well as exert the advantages of multiphoton imaging.

In this work, the upconversion emission mechanisms of Tm^3+^-doped nanoparticles under excitations of different NIR-II lasers were systematically investigated and compared. The 1064 nm, 1150 nm, and 1208 nm lasers are proposed to be three NIR-II excitation strategies that can generate different emission spectra from Tm^3+^ ions. In addition, we excavated the mechanism of energy quenching and further optimized the structure of upconversion nanoparticles to alleviate the luminescence quenching. The high-brightness NIR-II-excitation Tm^3+^-doped nanoparticles enable great performance upconversion laser scanning microscopic imaging for nanoparticles and cancer cells.

## 2. Materials and Experimental Methods

### 2.1. Materials

Thulium(III) acetate hydrate (99.9%), ytterbium(III) acetate hydrate (99.9%), yttrium(III) acetate hydrate (99.9%), and N-(3-Dimethylaminopropyl)-N′-ethylcarbodiimide hydrochloride (EDC, ≥98%) were purchased from Sigma-Aldrich (Steinheim, Germany). Sodium hydroxide (NaOH, ≥98%), ammonium fluoride (NH_4_F, ≥99.99%), 1-octadecene (ODE, ≥90% (GC)), oleic acid (OA, AR), cyclohexane (AR, 99.5%), Nitrosyl tetrafluoroborate (NOBF_4_, 95%), and N, N-Dimethylformamide (DMF, 99.5%) were purchased from Aladdin Reagent Company (Shanghai, China). Ethanol (AR), methanol (AR), ethanol (AR), and dimethylsulfoxide (DMSO, AR) were purchased from Sinopharm Chemical Reagent Co. (Beijing, China). 2-(N-Morpholino) ethanesulfonic (MES) acid Buffer (0.1 M, pH = 6.0) was purchased from Leagene Biotechnology co. (Beijing, China). N-Hydroxysuccinimide (NHS, 98%), Phosphate-buffered Saline (PBS), and ProLong™ Gold Antifade Mountant were purchased from Thermo Fisher Scientific (Waltham, MA, USA). Triton X-100 was purchased from Amresco (Solon, OH, USA). Quick Block™ blocking buffer and Quick Block™ primary antibody dilution buffer for immunol staining were purchased from Beyotime (Beijing, China). The primary antibody (anti-desmin monoclonal antibody, Y66, ab32362) was purchased from Abcam (Cambridge, UK), and the secondary antibody (Goat Anti-rabbit IgG, bs-0295G) was purchased from Bioss (Beijing, China). Cell Counting Kit-8 (CCK-8) was purchased from Meilunbio (Dalian, China). All the reagents were used as received without further purification.

### 2.2. Synthesis of NaYF_4_: Tm@NaYF_4_ Core-Shell Nanoparticles

The singly doped NaYF_4_: Tm@NaYF_4_ core-shell nanoparticles and the co-doped NaYF_4_: Yb/Tm@NaYF_4_ core-shell nanoparticles were synthesized using previous reported co-precipitation strategy with modifications [15]. In a 100 mL bottom flask, 5 mL Ln(CH_3_CO_2_)_3_ (Ln = Tm/Y/Yb, the adding proportion depends on the doping ratio) stock solution (0.2 M) was added to 7.5 mL OA and 17.5 mL ODE. The mixture was heated up to 150 °C and kept for 40 min to form the lanthanide-oleate precursor solution, and then was cooled down to room temperature. 2.5 mL NaOH-methanol stock solution (1 M) and 10 mL NH_4_F-methanol stock solution (0.4 M) were added into the flask, and then the mixture was kept at 40 °C for an additional 2 h. Subsequently, the mixture was heated to 100 °C for 30 min under vacuum to evaporate the methanol. In an argon atmosphere, the mixture was further elevated to 310 °C and kept for 1.5 h. After reaction, the solution was cooled down to room temperature and washed with 15 mL ethanol, centrifuged at 7500 r.p.m for 5 min. The obtained core nanoparticles were then washed with ethanol and cyclohexane three times and re-dispersed in 8 mL cyclohexane for the subsequent epitaxial growth of the inert NaYF_4_ shell layer.

Wrapping an inert NaYF_4_ shell on the core nanoparticles follows a similar synthesis procedure. To the 100 mL bottom flask, 5 mL Y (CH_3_CO_2_)_3_ stock solution (0.2 M) with 7.5 mL OA and 17.5 mL ODE were added. The mixture was also heated to 150 °C and kept for 40 min, then was cooled down to room temperature. Before adding the 2.5 mL NaOH-methanol stock solution and 10 mL NH_4_F-methanol stock solution, 4 mL core nanoparticles suspension was injected into the flask, then the subsequent steps were the same. The obtained core-shell nanoparticles were also re-dispersed and stored in 8 mL cyclohexane. The concentration of this as-prepared core-shell UCNPs suspension is 15 mg mL^−1^.

### 2.3. Hydrophilic and Surface Functionalization of the UCNPs

The ligand-exchange process of nanoparticles was prepared using previously reported methods [16]. Typically, 5 mL DMF and 50 mg NOBF_4_ were added into 5 mL cyclohexane containing 0.1 mmol as-prepared nanoparticles and vigorously stirred for 5 min. The as-prepared nanoparticles were precipitated by adding an excess of toluene and collected by centrifugation at 14,000 r.p.m. for 30 min. Discarding the supernatant, excess DMF/toluene (1:1) mixed solution was added to the centrifuge tube, colorless transparent precipitate was obtained by centrifugation at 14,000 r.p.m. for 30 min. The product was re-dispersed into 1 mL deionized water, and added into 400 μL PAA solution (1.5 wt.%), which dissolved in 11.6 mL deionized water drop by drop. The mixture was stirred over 2 h and centrifugation was performed at 15,000 r.p.m. for 20 min. The precipitates were re-dispersed in water to obtain a colorless and transparent colloidal solution. The carboxyl groups on the surface of UCNPs were activated and modified according to the usual methods [17,18]. Dissolving EDC and NHS solids in MES solution and controlled the concentration to 0.2 and 0.3 mg μL^−1^, respectively. After adding 20 μL each of the NHS and EDC solutions of the set concentration into the prepared 1 mL PAA-UCNPs aqueous solution, the mixture was stirred at room temperature for 2 h. After this, the UCNPs were acquired by centrifugation at 15,000 r.p.m. for 30 min and re-dispersed in 1 mL ultrapure water. Subsequently, the pH of the solution was adjusted to slightly alkaline to ensure that UCNPs activated by NHS can effectively react with antibody. After adding 500 μg of diluted goat antirabbit antibody IgG, the mixture was stored in a refrigerator at 4 °C overnight, and then centrifuged at 14,000 r.p.m for 20 min in the next day. The obtained sediment at the bottom of the centrifuge tube was redispersed in PBS buffer for later use.

### 2.4. Cell Culture and Immunolabelling

A commonly used immunostaining technique was used to perform cancer cell labeling using UCNPs [17,19]. The HeLa cells were cultured overnight in a 96-well confocal plate, with approximately 15,000 cells in each unit. In the second day, the cells were rinsed with PBS buffer and fixed using immunostaining fixative for 20 min at room temperature. The fixed cells were washed and permeabilized with immunostaining 0.2% Triton X-100 for 20 min. Washing with PBS buffer followed by the addition of Quick Block blocking solution for 20 min. Using Quick Block primary antibody Dilution buffer to prepare antibody dilutions. The primary antibody (anti-desmin monoclonal antibody) solution of 5 µg mL^−1^ was used to incubate the fixed cells at 4 °C overnight and rinsed three times using PBS buffer. Incubating the fixed cells at 4 °C overnight with 5 µg mL^−1^ of the primary antibody (anti-desmin monoclonal antibody) solution, and washed 3 times with PBS buffer. Then, the as-prepared UCNPs bioconjugated with secondary antibodies were dissolved in PBS buffer after centrifugation. Subsequently, after centrifugation, the as-prepared UCNPs biologically coupled with the secondary antibody were dissolved in PBS buffer. A volume of 100 μL UCNPs solution (200 µg mL^−1^) was added to 96-well plates with the primary antibody-treated fixed cells. Adding 100 μL of UCNPs solution (200 μg mL^−1^) to the fixed cells treated with primary antibody in a 96-well plate. The immunostaining reaction of cancer cell was kept for 2 h at room temperature. The cells were then washed with pre-warmed (37 °C) PBS buffer twice for later imaging.

### 2.5. Cytotoxicity of Surface-Modified UCNPs

The cell viability of Hela cells after treatment with PAA-UCNPs mentioned before were evaluated by CCK-8 assay. Briefly, the HeLa cells were cultured overnight in a 96-well confocal plate for attachment, with approximately 15,000 cells in each unit. The next day, replaced the overnight medium in the 96-well confocal plate with fresh medium which contained various concentrations of PAA-UCNPs (10–500 μg mL^−1^) and the cells were incubated at 37 °C under 5% CO_2_ for 4 h. After that, 10 μL of CCK-8 (10 mg mL^−1^) was added to each unit subsequently and the cells were continue incubated in the same condition for 1.5 h. The cell viability was calculated by measuring the absorption of CCK-8 at 450 nm using a 400–750 nm microplate reader (BIO-RAD imark™).

## 3. Characterization Methods

The transmission electron microscopy (TEM) images were obtained using a high-resolution transmission electron microscope (JEM-2100HR, JEOL (200 kV), Tokyo, Japan). All the samples were diluted and dispersed in cyclohexane, then dropped onto the surface of copper grids for the test of TEM.

### Optical Setup for Spectroscopic and Microscopic Studies

The spectroscopic measurement and fluorescence imaging were performed in a lab-made optical system. The 1064 nm, 1150 nm, 1208 nm, and 980 nm pulse laser beam were, respectively, generated by a single-mode diode laser (Changchun New Industries Optoelectronics Tech. Co., Ltd. (Changchun, China), Shenzhen Leo-Photoelectric Co., Ltd. (Shenzhen, China) and Shanghai B&A Technology Co. Ltd. (Shanghai, China)). These laser beams were directed into a multiphoton laser scanning microscope (IX81, Olympus, Tokyo, Japan), where laser beams were reflected by a 950 nm short-pass dichroic mirror (Chroma) and then focused on the samples (dropped on glass slides) through an oil-immersed objective (OL, PL-APO × 60/1.35, Olympus, Tokyo, Japan). The upconversion emission was collected by the same objective and passed through the dichroic mirror. Then, a controllable switch in microscope determined the emissions to be detected by a fiber-based spectrometer (QE65000, Ocean Optics, Dunedin, FL, USA) for spectroscopic measurement, or by a pair of photomultiplier tubes (PMT) for fluorescence imaging and lifetime measurement. A dichroic mirror (ZT458rdc, Chroma) combined with band-pass filter (ET480/20x, Chroma) and (ET440/40x, Chroma) was used to select the three-photon 475 nm emission band and the four-photon 455 nm emission band. For lifetime measurement, a chopper (model SR540, Stanford) was set on the optical path and used to modulate the excitation beam. The trigger signal from the chopper and the detected single photons by PMT were synchronized with a time-correlated single-photon counter (NanoHarp, PicoQuant, Berlin, Germany). The modulation frequency was set to be 1 kHz.

## 4. Results

**Figure 1:**
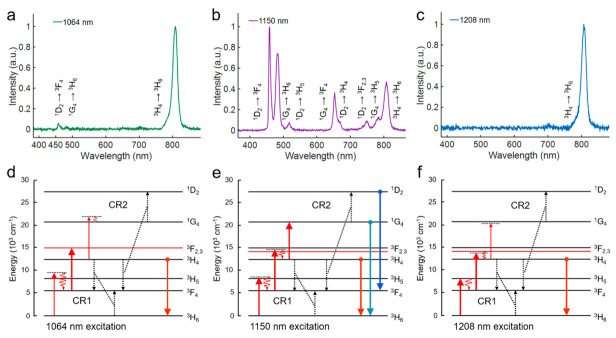
(**a**–**c**) Upconversion emission spectra of NaYF_4_: Tm (1.75%) UCNPs excited at 1064 nm, 1150 nm, 1208 nm lasers, respectively. The intensities of all the excitation wavelengths were kept at 1.5 MW cm^−2^. (**d**–**f**) Proposed excitation mechanisms in Tm^3+^ doped UCNPs under the 1064 nm, 1150 nm, and 1208 nm lasers excitation.

To systematically study the upconversion mechanism of the three NIR-II excitation routes for Tm^3+^ ions, we firstly analyzed and compared the emission characteristics. As shown in Figure 1a–c, a series of upconversion emission bands of Tm^3+^ ions were observed under all the three NIR-II excitations. Interestingly, their spectra are quite different from each other, indicating there are different mechanisms involved in these NIR-II excitation processes. When the nanoparticles were excited by the 1064 nm laser, the emission band at 808 nm (^3^ H_4_ → ^3^ H_6_) was dominant in the upconversion spectrum, while the higher-order blue emission at 475 nm (^1^ G_4_ → ^3^ H_6_) and 455 nm (^1^ D_2_ → ^3^ F_4_) were extremely weak. The ratio between 808 nm and 475 nm was about 147, implying the non-resonant excitation of 1064 nm laser to the higher-lying states ^1^ G_4_ and ^1^ D_2_. Similarly, for 1208 nm excitation, the upcoversion spectrum was also characteristic with a single strong emission band centered at 808 nm, but with a lower intensity than that generated by 1064 nm excitation, and the 455 nm emission could not be observed. Interestingly, for the case of 1150 nm excitation, besides the 808 nm emission band, three main emission bands at 475 nm, 455 nm, and 650 nm (^1^ G_4_ → ^3^ F_4_) together with several weak emission bands at 750 nm (^1^ D_2_ → ^3^ F_2,3_) and 785 nm (^1^ G_4_ → ^3^ H_5_) were generated. Moreover, the blue emission at 475 nm from higher-lying states was 1.6 times stronger than that at 808 nm. The intensities of the high-order blue emission at 475 nm and 455 nm under 1150 nm excitation were 100 times and 15 times stronger than that under the 1064 nm excitation. These results evidenced the superiority of the 1150 nm excitation for the Tm^3+^-doped nanoparticles.

The different intensity ratios between higher-order and lower-order upconversion emission bands under different excitation routes are correlated to the energy distribution discrepancy among the energy states, which are mainly caused by the CR process. To determine the upconversion mechanism, it is necessary to analyze the energy transfer characteristic of CR involved in the NIR-II excitation system. Under 1064 nm excitation, the strong 808 nm emission generated by 1064 nm laser can be mainly attributed to an energy-looping process [14], consisting of an excited state absorption (ESA) process (^3^ F_4_ → ^3^ F_2_) and a CR process (CR1, ^3^ H_4_ + ^3^ H_6_ → ^3^ F_4_ + ^3^ F_4_) (Figure 1d). In this process, since the non-resonant GSA process (^3^ H_6_ → ^3^ H_5_) could only provide few electrons for the subsequent ESA, the CR1 process here plays an important role in lifting the electrons from the ground state to the intermediate state.

**Figure 2:**
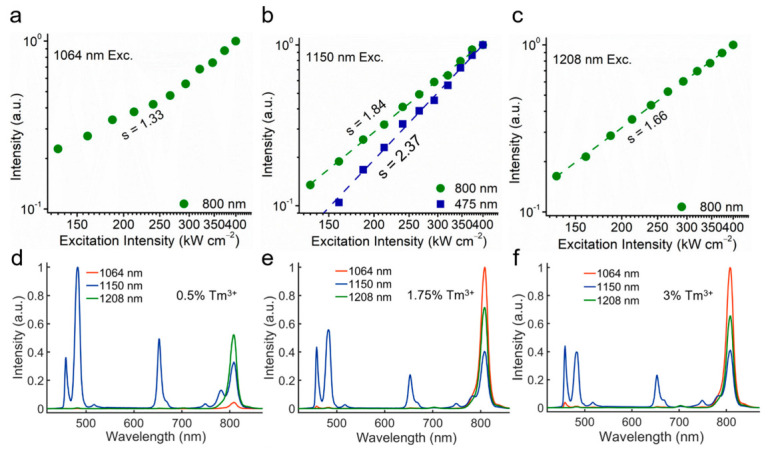
(**a**–**c**) Dependence of the emission intensities of NaYF_4_: Tm (1.75%) sample on excitation intensity at 1064 nm, 1150 nm, and 1208 nm, respectively. (**d**–**f**) Spectra of NaYF_4_: Tm (0.5/1.75/3%) samples excited by NIR-II lasers at the intensity of 1.5 MW cm^−2^.

The spectra results (Figure 2d–f) of different concentrations of Tm^3+^ ions at different emission wavelengths show that, when the Tm^3+^ concentration decreases to 0.5%, the CR1 process is less effective, and the originally strongest 808 nm emission excited by 1064 nm becomes even weaker than that excited by 1150 nm and 1208 nm. This phenomenon reveals that the excitation processes of 1150 nm and 1208 nm are less dependent on the CR1 because of the better matching between the ground state absorption (GSA) transition and the incident photon energy. In addition, the blue emissions excited by 1150 nm and by 1064 nm also show a dependence on the Tm^3+^ concentration, first increasing and then decreasing. The CR process (CR2, ^1^ G_4_ + ^3^ H_4_ → ^1^ D_2_ + ^3^ F_4_) (Figure 1e) is still the major route for the population of ^1^ D_2_ state rather than a direct ESA process. Indeed, although the 1150 nm excitation process shows less dependence on the CR1, the effect of CR1 would still contribute to the enhancement of 808 nm emission and trap electrons in the lower-lying states as the case of 1064 nm excitation, which lowers the ratio between the higher- and lower-order emissions. Thereby, choosing a moderate concentration of Tm (1.75%) can avoid excessive CR effect of the higher concentration of activator to maintain high emission intensity.

To determine the photon number absorbed in these NIR-II excited upconversion processes, we then measured the dependence curves of major emission bands on the excitation intensity. As shown in Figure 2a–c, when excited by three different NIR-II wavelengths, the slopes of the 808 nm emission band are 1.33, 1.84, and 1.66, respectively. Due to the possible saturated excitation processes, the measured slope would be always lower than the expected number of pump photon. It can be determined that the ^3^ H_4_ state is excited by similar two-photon processes for all these three NIR-II excitation cases. In the case of 1150 nm excitation, the 475 nm emission with the slope at 2.37 indicates that there are three-photon processes involved in the excitation of the ^1^ G_4_ states.

Based on the above experimental observation and analyses, now we can distinguish the excitation mechanisms of these three NIR-II wavelengths. As depicted in Figure 1d–f, the GSA process: ^3^ H_6_ → ^3^ H_5_ and the following two-steps ESA processes: ^3^ F_4_ → ^3^ F_2,3_ and ^3^ H_4_ → ^1^ G_4_ are the common transition pathways for all three excitation strategies. The reason for the difference in emissions is mainly the energy mismatching between the energy gaps of these three upconversion transitions and the incident photon energy. Specifically, the 1064 nm laser matches well with the energy gap between ^3^ F_4_ and ^3^ F_2_, but there are relatively large mismatches in the other two transitions. Thus the 1064 nm excitation is a CR-based energy-looping process and is highly dependent on the doping concentration. The 1208 nm laser can directly pump the electrons from ground state ^3^ H_6_ to ^3^ H_5_. However, as for the second step, the relatively lower photon energy of 1208 nm cannot make the electrons at ^3^ F_4_ state suitably reach the ^3^ F_3_ state (note that the 1064 nm pumps the electrons to the ^3^ F_2_ state), which results in the lower intensity of 808 nm emission than that of 1064 nm. Similarly, the 1208 nm laser cannot generate strong higher-order blue emissions, also due to the mismatch in the transition ^3^ H_4_ → ^1^ G_4_. In contrast to the above two wavelengths, the excitation of 1150 nm could be regarded as a compromise method to simultaneously match the first two transitions. More importantly, the 1150 nm laser is the only one matching the energy gap between ^3^ H_4_ and ^1^ G_4_, which means that the electrons at ^3^ H_4_ can be further lifted to the higher-lying states for generating the higher-order blue emissions. Moreover, different from the traditional energy transfer upconversion (ETU) system where energy mismatching can be overcome with the assistance of phonon energy, the perfectly matched transition ^3^ H_4_ → ^1^ G_4_ here has an extra effect on regulating the distribution of electrons. Under this strong ESA process, the electrons were forced to transfer to the higher-lying states and tend to contribute to the higher-order emissions. Beyond that, the CR1 and CR2 processes mentioned above are involved in every NIR-II upconversion system, and the fourth-step upconversion to the ^1^ D_2_ state is mainly achieved by the CR2. In conclusion, among these three wavelengths, the 1150 nm upconversion system is the unique one that can combine the advantages of NIR-II wavelength and the higher-order nonlinear excitation, which make it an ideal UCNPs-based excitation strategy for the following researches of nanoparticles and cancer cell imaging.

**Figure 3:**
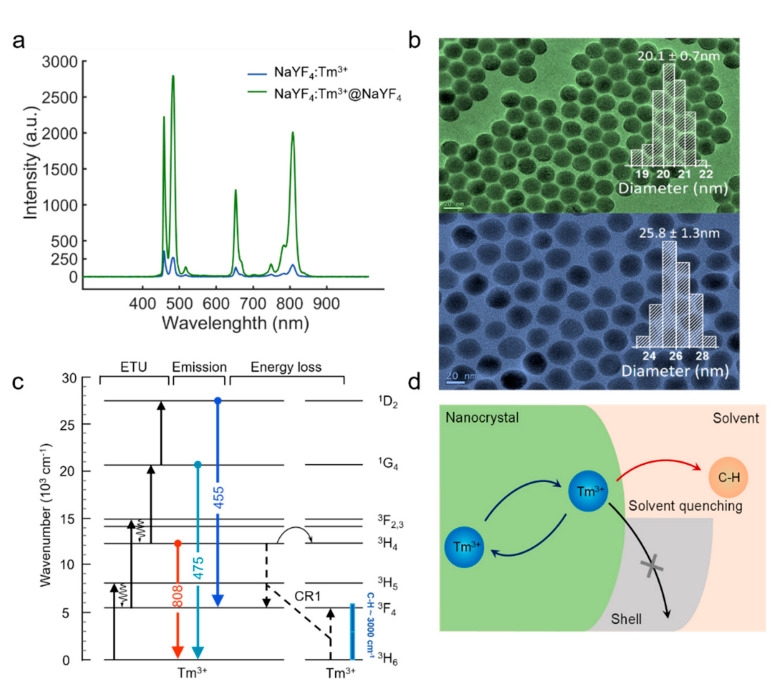
(**a**) Spectra of bare core NaYF_4_: Tm (1.75%) and NaYF_4_: Tm (1.75%)@NaYF_4_ core-shell samples excited by 1150 nm lasers at the intensity of 1.5 MW cm^−2^ (the concentration of both bare core and core-shell UCNPs samples are 150 μg mL^−1^). (**b**) TEM image of the as-prepared core NaYF_4_: Tm (1.75%), average size: 20.1 ± 0.7 nm in diameter. TEM image of the as-prepared core-shell NaYF_4_: Tm (1.75%)@NaYF_4_ UCNPs, average size: 25.8 ± 1.3 nm in diameter. (**c**) Energy diagram scheme of Tm^3+^ ion and proposed energy transfer pathways for upconversion. (**d**) Schematic of solvent quenching, where an excited state on a Tm^3+^ lanthanide center doped in a nanocrystal can decay by transferring energy to high energy vibrations in the surrounding solvent.

Fluorescence imaging has high brightness requirements for UNCPs. The luminescence properties especially the upconversion efficiency of UCNPs are very sensitive to variations in the surface area-to-volume ratio, crystal structure, and lanthanide doping concentration as well as to the ligand and surrounding medium [20]. Previous study about Yb^3+^ sensitizer-activator system indicates that energy attenuation of the sensitizer caused by surface defects is the primary reason for the decrease of fluorescence intensity. However, the studies about the surface quenching effect on the single-doped Tm^3+^ system excites by NIR-II wavelengths is unspecified. By comparing the same dilution concentration of NaYF_4_: Tm (1.75%) core-only nanoparticles with the core-shell NaYF_4_: Tm (1.75%)@NaYF_4_ nanoparticles, the luminescence intensity of the core-shell sample is nearly ten times higher than the former one (Figure 3a). This phenomenon indicates that severe luminescence quenching effect induced by surface defects still exists in singly doped Tm^3+^ UCNPs. Therefore, the mechanism that causes the energy loss of the bare core structure can be inferred.

As evidenced by the effect of energy resonances between electronic transitions of the lanthanide ions and vibrations of the solvent molecules [21]. The energy level diagram of the core–shell nanoparticles NaYF_4_: Tm (1.75%)@NaYF_4_ is shown in Figure 3c. Here, the solvent is cyclohexane, and the highest-energy vibrational modes in hydrocarbon molecules are C–H stretch modes with energies around 3000 cm^−1^. Resonances of the C–H stretch vibrational energy with transitions between the Tm^3+^ states will directly affect the strength of solvent quenching [22]. The near-infrared levels ^3^ F_4_ can be quenched by coupling to the C–H stretch vibrational energy (Figure 3d) via overtone vibrational transitions, since the gap of the two energy levels (^3^ F_4_ → ^3^ H_6_) of Tm^3+^ (~5577 cm^−1^) is nearly 2 times of the highest solvent vibrations (C–H stretch) [23]. In addition, the effect of CR1 will drive the electrons to tend to concentrate at the ^3^ F_4_. In this context, the Tm^3+ 3^ F_4_ level would be easily quenched by the solvent, and thereby affects the emission intensity of the nanoparticles NaYF_4_: Tm (1.75%). Furthermore, CR as an energy transfer progress by dipole−dipole interaction depends on the distance between a donor center and an acceptor center, in which a luminescent center transfers part of its energy to neighbors, and finally to the surface quenchers [22,24]. To address the fluorescence quenching effect, we coated the core NaYF_4_: Tm (1.75%) with an inert shell NaYF_4_. Core-shell nanostructures are believed to confine energy migration and prevent energy trapping by quenching centers.

**Figure 4:**
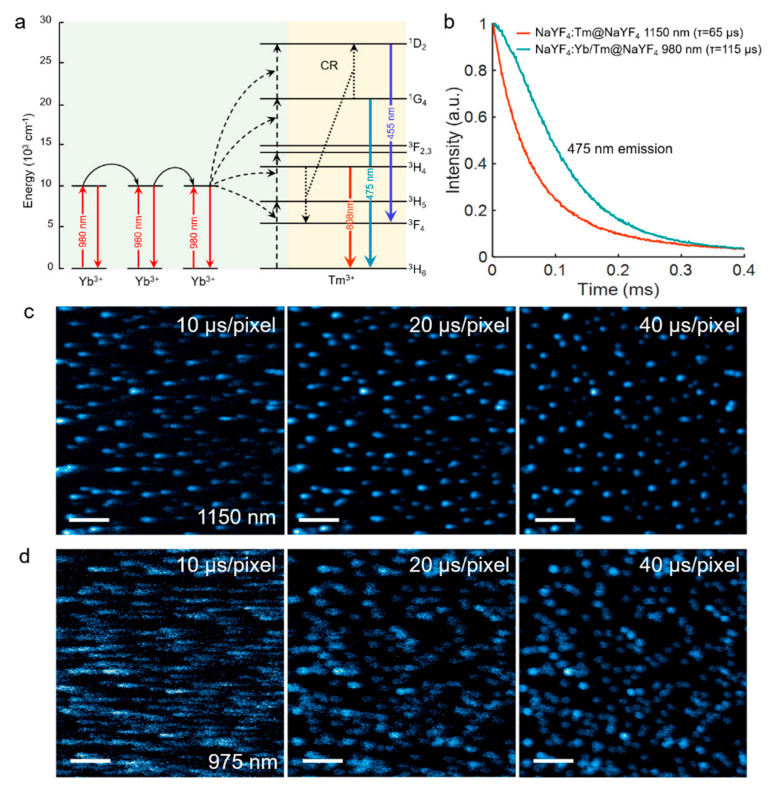
(**a**) Upon excitation of Yb^3+^ at 980 nm, three consecutive energy transfer processes (dotted lines) excite an Tm^3+^ ion to a high excited state. Subsequently, after one or more nonradiative decay steps Tm^3+^ can emit a visible photon. Other, more complicated, sequences of processes also contribute to the population of visible-emitting states. (**b**) Decay lifetimes of the 475 nm emissions of the NaYF_4_: Tm (1.75%)@NaYF_4_ UCNPs and NaYF_4_: Yb/Tm (18/1.75%)@NaYF_4_ UCNPs irradiated by 1150 nm and 980 nm excitation beams, respectively. (**c**,**d**) Multiphoton laser scanning luminescence images of the 475 nm emissions of the two samples, scale bar: 4 μm. The dwelling times were successively set to be 10, 20, and 40 μs/pixel. The resolution of these images is 512 × 512 pixels.

In Figure 4a, our work also studied the energy transfer path of traditional Yb^3+^/Tm^3+^ co-doped system. Through successive energy transfer steps from three (or more) Yb^3+^ ions to Tm^3+^, the conversion process from low-energy near-infrared photons to high-energy visible photons can be realized. In principle, the decay time extracted from the upconversion luminescence decay profile generally cannot be interpreted as the intrinsic lifetime of the upconversion luminescence emitting state [25]. Instead, it is an overall temporal response of the whole upconversion system to the excitation function, influenced by the sensitizer’s excited-state intrinsic lifetime and the effects of energy transfer [25]. Since these NIR-II excited upconversion processes do not rely on the Yb^3+^ sensitizer with long-lived state, the upconversion luminescence decay would not be influenced by the long excited-state lifetime of the sensitizers and the effect of energy transfer. Therefore, compared to traditional 980 nm excited Yb^3+^/Tm^3+^ co-doped system, shorter decay lifetimes of three-photon emissions are also expected in this 1150 nm upconversion system. As the decay profiles of 475 nm emissions of different upconversion systems shown in Figure 4b, it is found that the 475 nm blue emissions aroused by the direct excitation of 1150 nm beam in singly doped Tm^3+^ sample exhibit faster kinetics than those by the excitation of 980 nm beam in Yb^3+^/Tm^3+^ co-doped sample. Specifically, the measured decay lifetime of 475 nm excited by 1150 nm beam (65 μs) is nearly half of that excited by 980 nm beam (115 μs), which can help to improve the imaging scanning speed and hold larger potential for the application of rapid, dynamics life studies [26]. We then compared the laser scanning imaging results of these two upconversion systems obtained with different imaging speed (Figure 4c,d). Clearly, when the scanning dwelling time was reduced from 40 μs/pixel to 10 μs/pixel, the 475 nm three-photon fluorescence images excited by 1150 nm beam only show a relatively slight smearing. The position of nanoparticles can still be easily determined from the luminescence spots. In contrast, the longer lifetime of the emission excited by 980 nm beam suffered more severe streaking effect during the imaging. The spots became streaky and would destroy the lateral resolution in the scanning direction. In this case, the imaging speed was restricted. It should be noticed that in traditional photon upconversion of Tm^3+^ system, through step-wised ETU processes assisted by Yb^3+^ ions, the higher-order blue emissions were always much weaker than the lower-order 808 emission because the electrons tended to populate at lower-lying states [27]. 

**Figure 5:**
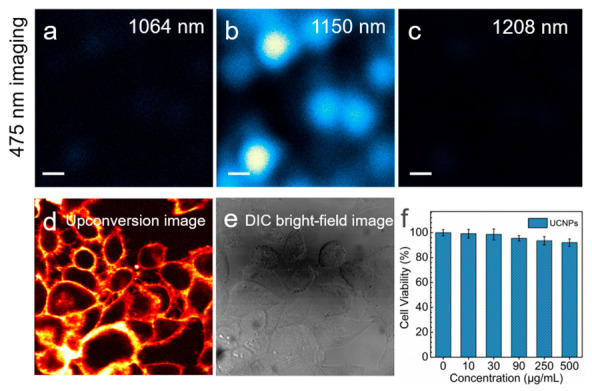
(**a**–**c**) Multiphoton scanning microscopic images of 475 nm emission of NaYF_4_: Tm (1.75%)@NaYF_4_ nanoparticles under 1064 nm, 1150 nm, 1208 nm laser excitation. The excitation intensities were kept at 3 MW cm^−2^. The sample slide distributed with nanoparticle was prepared by spin-coating method. Scale bar: 500 nm. (**d**) Upconversion image of HeLa cells after treatment with NaYF_4_: Tm (1.75%)@NaYF_4_ UCNPs. Micrograph of cell fluorescence excited at 475 nm following imaging over 85 s at 1150 nm. The resolution of these images is 640 × 640 pixels. (**e**) Differential Interference Contrast (DIC) image. (**f**) Viability of Hela cell incubated with different concentrations of PAA-coupled NaYF_4_: Tm (1.75%)@NaYF_4_ UCNPs for 4 h.

As mentioned above, 808 nm is in a luminescence band where the detector cannot respond sensitively. From the combination of excitation mechanisms and spectrogram results, 1150 nm is the most favorable wavelength for blue emission, which is more conducive to subsequent luminescence decay lifetime, nanoparticles, and cancer cell imaging research. The excellent performance of 1150 nm excitation strategy was also demonstrated through multiphoton scanning microscopic imaging. The bright blue 475 nm luminescence excited by 1150 nm laser was successfully observed from the nanoparticles. In contrast, the 1064 nm and 1208 nm excitation can only use the 808 nm emission for imaging (Figure 5a–c). Hela cells labeled with the hydrophilic and surface functionalization UCNPs (20 nm diameter; 1.75% Tm^3+^) with 5 nm inert shell have been manipulated with 1150 nm lasers as cancer cell fluorescence imaging (Figure 5d,e). For upconversion imaging, excitation at NIR-II wavelengths and anti-Stokes NIR emission suggests the possibility of imaging with little or no phototoxicity or autofluorescence background. We sought to demonstrate that UCNPs could be deployed and unambiguously visualized inside cells with 1150 nm excitation. We also assessed the cytotoxicity of the UCNPs by CCK-8 assay. As shown in Figure 5f, the cell viability was calculated to be larger than 92% even at a concentration as high as 500 μg mL^−1^ PAA-coupled NaYF_4_: Tm (1.75%)@NaYF_4_ UCNPs, which indicated the UCNPs exhibited good biocompatibility and nearly nontoxic. The cytotoxicity and bioimaging results illustrated the UCNPs as biological labels can achieve a well presentation of the cellular morphology and contour. The bright-field morphology corresponding to the fluorescence labeled cells is shown in Figure 5e. 

## 5. Conclusions

In the work, by studying and comparing the mechanisms of Tm^3+^-doped UCNPs excited by three NIR-II excitation strategies of at 1064 nm, 1150 nm, and 1208 nm, we found that only the photon energy of 1150 nm can match well with the energy gaps between the ^3^ H_4_ and ^1^ G_4_ states and populate the higher-lying states for generating the three and four-photon blue emissions, while the 1064 nm and 1208 nm could only effectively generate a two-photon 808 nm emission. We also investigated the mechanism of the effect on luminescence quenching on NIR-II wavelength excitation in detail, then pointed out that the solvent quenching effect, energy transfer and CR processes would affect the upconversion emission in Tm^3+^-doped system. In the long term, this work possessed far-reaching significance for enriching and deepening the upconversion luminescence mechanism. By combining the advantages of NIR-II wavelength excitation and higher-order nonlinear excitation process, this proposed 1150 nm excitation strategy holds a great potential to perform nanoparticles and nontoxic cancer cell microscopic imaging with high signal-to-background ratio, high-resolution and easily accessible laser source. The reason why we chose the pulsed laser is for ease to modulate the excitation wavelength. However, in fact, the current experimental results are also achievable excited by the continuous-wave lasers. We believe our work will give a new understanding to the NIR-II excited nanoprobes and provide a theoretic basis for subsequent NIR-II bioimaging applications.

## Data Availability

Not applicable.
